# ANCA-MPO: is this a useful test?

**DOI:** 10.1093/rheumatology/kead173

**Published:** 2023-05-03

**Authors:** Ana Mafalda Abrantes, Lorena Montaño-Tapia, David Isenberg

**Affiliations:** Division of Internal Medicine II, Centro Hospitalar Universitário Lisboa Norte, Lisbon, Portugal; Instituto de Semiótica Clínica, Faculty of Medicine, University of Lisbon, Lisbon, Portugal; Rheumatology Department, Príncipe de Asturias University Hospital, Alcalá de Henares, Madrid, Spain; Department of Medicine, Centre for Rheumatology, University College London, London, UK

Rheumatology key message

- ANCA-MPO may not accurately predict ANCA-associated vasculitis and drug-induced vasculitis.

Dear Editor, ANCAs bind proteins expressed in neutrophils responsible for vascular inflammation [1]. ANCA-associated vasculitis (AAV) is a small-vessel vasculitis characterized by necrotizing vasculitis, with few/absent immune deposits. This classification includes granulomatosis with polyangiitis (GPA), microscopic polyangiitis (MPA), and eosinophilic granulomatosis with polyangiitis (EGPA) [2].

Positive staining of ANCA has three main patterns by IIF: p-ANCA, c-ANCA, and atypical. The last includes all the other neutrophil or monocyte-specific immunofluorescent reactivity, resulting from a mixture of cytoplasmic and perinuclear staining that can be present in other inflammatory diseases [3]. The immunoassay detects antibodies against PR3 and MPO, which are strongly associated with c-ANCA and p-ANCA, respectively [2].

We revisited the question of the usefulness of the ANCA-MPO assay and, in particular, whether it is of value in diagnosing AAV and drug-induced vasculitis. We performed a retrospective analysis of patients whose ANCA-MPO test was performed between July and December 2022 at University College London Hospital. We defined the following secondary goals: (a) assess which specialties most frequently request this test; (b) identify the diagnoses made; (c) determine the frequency of positive ANCA-MPO tests; (d) evaluate whether the ANCA screen test, including the atypical p-ANCA and p-ANCA patterns, correlated with the ANCA-MPO test. The principles of the Declaration of Helsinki were observed during the study period.

ANCA-MPO test was performed on 614 patients: 64.8% females and 35.2% males. Of the patients whose ethnicity was confirmed, Caucasians constituted 54.4%, Asians 13.2% and Afro-Caribbeans 13.2%. Rheumatology (38.1%), Neurology (17.4%), Respiratory (6.5%), Dermatology (6.4%), Nephrology (5.5%) and ENT (5.4%) were the specialties who most frequently requested the test. Forty patients had a diagnosis of AAV, or cocaine-induced vasculitis. We also noted LN (4.3%), RA (3.7%), SLE (3.5%), and interstitial lung disease (2.8%). No diagnosis had been reached in 18.6%. Clinical diagnosis was inconclusive in 2.9%. GPA was established in 2.4%, EGPA in 0.9%, AAV in 1.6%, and cocaine-induced vasculitis in 0.4%. No patients tested had a diagnosis of MPA.

The ANCA screen test, performed in 476 patients (77.5%) was positive in 43.2%. Among them, 25.7% and 23.4% showed a p-ANCA and atypical p-ANCA pattern, respectively. The most frequent pattern was atypical c-ANCA (29.8%). The ANCA-MPO test was positive in 2.0%.

The performance of the ANCA-MPO test as a predictor of AAV or drug-induced vasculitis was assessed through receiver operating characteristic curve analysis. The area under the curve (AUC) was 0.59 (95% CI 0.5, 0.7). The Youden index was determined to establish the cut-off value with the highest validity of the ANCA MPO test. The optimal cut-off was 0.3, with a sensitivity of 40.0% and a specificity of 79.0% (Youden index 0.2). Calibration was verified through the Hosmer–Lemeshow test. The ANCA screen result did not correlate with the ANCA-MPO result [odds ratio (OR) 7.4; *P*-value 0.059; 95% CI 0.9, 58.7]. No correlation was also identified with p-ANCA (OR 3.8; *P*-value 0.05, 95% CI 0.1, 14.7) or atypical p-ANCA (OR 0.9; *P*-value 0.933; 95% CI 0.2, 4.6).

AAV is uncommon, with diverse clinical features [4]. Guchelaar *et al.* [5] compared the diagnostic value of ANCA IIF and immunoassay for diagnosing AAV. The sensitivity obtained for ANCA-MPO with immunoassay was 58.1%, and the specificity was 95.6%. Although the ANCA test is considered relevant for AAV diagnosis, our data showed the discriminatory ability of the test is unsatisfactory, as the AUC is <0.6. With a Youden index close to zero, the ANCA-MPO test showed little ability to discriminate between patients with AAV and those with drug-induced vasculitis. Given the low sensitivity of the test, the percentage of undetected cases is relatively high. Furthermore, the ANCA screening results were not correlated with the ANCA-MPO result, confirming the superior immunoassay sensitivity, and raising awareness about the uncertain clinical significance of an ANCA screen test.

Specialties who requested the test most frequently were those managing the common symptoms of AAV were, renal, pulmonary, including upper respiratory tract, and dermatological features.

In addition to AAV and drug-induced vasculitis, 200 additional diagnoses were noted among those for whom the test was requested (see Table 1). Our study supports previous data describing a positive ANCA test in non-vasculitic conditions. This is due to indiscriminate testing in patients with low pre-test probability of AAV or drug-induced vasculitis, increasing the frequency of false-positive results obtained. Although the 2017 consensus statement [6] recommends testing with high quality MPO-ANCA assays rather than screening with IIF, this has serious cost implications. We suggest that internal policies should guide the use of this test so that it is only requested when clinically appropriate [7].

**Table 1.** Patients’ demographic, clinical and laboratory data. Data are shown as number (%) for categorical variables and median ± interquartile range for continuous variables. The denominators of patients who were included in the analysis are provided if they differed from the overall numbers within the group

Characteristics	Total (*n* = 614)
**Age (year)**	48.98 ± 18.04
**Gender**	
Female	398 (64.8)
**Ethnicity**	
Caucasian	230 (37.5)
Asian	56 (9.1)
African	56 (9.1)
Mixed ethnicity	8 (1.3)
Other ethnicity	73 (11.9)
Unknown	191 (31.1)
**Specialties that required the test**	
Rheumatology	234 (38.1)
Neurology	107 (17.4)
Respiratory medicine	40 (6.5)
Dermatology	39 (6.4)
Nephrology	34 (5.5)
Ear, nose and throat	33 (5.4)
Haematology	27 (4.4)
Gastroenterology	26 (4.2)
Accident and emergency	10 (1.6)
Geriatric medicine	7 (1.1)
Acute medicine	6 (1.0)
Oncology	6 (1.0)
Infectious diseases	6 (1.0)
Pharmacology	5 (0.8)
Obstetrics	4 (0.7)
Audiovestibular medicine	3 (0.5)
Tropical medicine	3 (0.5)
Critical care medicine	3 (0.5)
General (internal) medicine	2 (0.3)
General practitioner	2 (0.3)
Orthopaedics	2 (0.3)
Allergy and clinical immunology	1 (0.2)
Cardiology	1 (0.2)
Endocrinology	1 (0.2)
Gynaecology	1 (0.2)
Neurogenetic	1 (0.2)
Neurosurgery	1 (0.2)
Oral medicine	1 (0.2)
Sport and exercise medicine	1 (0.2)
Vascular surgery	1 (0.2)
Unknown	6 (1.0)
**ANCA screen**	
Positive	265 (43.2)
Negative	211 (34.4)
Not requested	138 (22.5)
**ANCA screen pattern**	
Atypical c-ANCA	79/265 (29.8)
p-ANCA pattern	68/265 (25.7)
Atypical p-ANCA	62/265 (23.4)
c-ANCA pattern	56/265 (21.1)
**Diagnosis**	
Rheumatologic diseases	223 (29.2)
Neurologic diseases	72 (9.4)
Respiratory medicine diseases	51 (6.7)
Nephrologic diseases	49 (6.4)
Gastroenterologic diseases	40 (5.2)
Haematologic diseases	37 (4.9)
Allergy and clinical immunologic diseases	21 (2.8)
Infectious diseases	20 (2.6)
Ear, nose and throat diseases	20 (2.6)
Oncologic diseases	14 (1.8)
Pharmacologic diseases	13 (1.7)
Dermatologic diseases	13 (1.7)
Orthopedic diseases	5 (0.7)
Ophthalmologic diseases	7 (0.9)
Cardiologic diseases	7 (0.9)
Vascular surgery diseases	3 (0.4)
Endocrinologic diseases	2 (0.3)
Obstetric diseases	1 (0.1)
Psychiatric diseases	1 (0.1)
Under investigation	142 (18.6)
Unknown	22 (2.9)
Mortality	13 (0.02)

The main limitations of our study are that it is single-centre and a retrospective study, but the detailed assessment of the clinical diagnosis in >600 patients in whom the test was performed does provide significant compensation.

In conclusion, our results indicate that the ability of ANCA-MPO to predict AAV or drug-induced vasculitis is limited.

## Data Availability

The authors would be happy to respond to any reasonable request to share the data in this manuscript.
